# Age-Dependent Face Detection and Face Categorization Performance

**DOI:** 10.1371/journal.pone.0079164

**Published:** 2013-10-08

**Authors:** Claus-Christian Carbon, Martina Grüter, Thomas Grüter

**Affiliations:** 1 University of Bamberg, Department of General Psychology and Methodology, Bamberg, Germany; 2 Graduate School of Affective and Cognitive Sciences, Bamberg, Germany; Tel Aviv University, Israel

## Abstract

Empirical studies on the development of face processing skills with age show inconsistent patterns concerning qualitative vs. quantitative changes over time or the age range for peak cognitive performance. In the present study, we tested the proficiency in face detection and face categorization with a large sample of participants (*N* = 312; age range: 2-88 yrs). As test objects, we used so-called Mooney faces, two-tone (black and white) images of faces lacking critical information of a local, featural and relational nature, reflecting difficult real world face processing conditions. We found that performance in the assessment of gender and age from Mooney faces increases up to about age 15, and decreases from 65 years on. The implications of these findings are discussed in the light of classic and recent findings from face development literature.

## Introduction

Visual face perception and processing in the human brain is generally believed to be an efficient and fast-running multistage process [1], though this view has recently been challenged by Rossion et al. [2] who see evidence for a non-hierarchical face perception process. Many studies have tried to clarify the mechanisms of face detection, processing, categorization (male/female, old/young, emotion, attractiveness), memory and recognition, but still, many details of the functional and anatomical foundations remain obscure (for an overview see [3]).

Several studies have shown that facial information processing involves the encoding of the shape and size of individual features like the eyes, nose and mouth (“featural” face processing, also called “analytic”, “componential” or “piecemeal”) as well as the encoding of the spatial relations and angles [4] between those features (“configural”, “configurational” or „relational“ face processing) [5,6] and the holistic quality of the face (“holistic” face processing) [7]—for a review and clarification of these terms see Piepers and Robbins [8].

The developmental changes in face *encoding* and *recognition* have been studied extensively (for a comprehensive overview see [9]). Face *detection* and face *categorization*, though, have not received as much attention. There is evidence that even newborns detect a frontally presented face [10] and may also be able to perform across view change [11]. This is quite remarkable because the features of a prototypical frontal face—two eyes above a nose in the middle above a mouth—no longer apply when then view is changed by angular deviations of 30° degrees or more implying that even newborns have a more sophisticated way of recognizing faces. Alternatively, they may learn that facial objects continuously change their aspect without losing their perceived and processed qualities.

Doi et al. [12] studied the detection abilities of Mooney faces in infants. In Mooney faces, named after Craig Mooney, who first used the faces to test visual closure (see [13]), graduated colour or grayscale information is dichotomized into white or black pixels. This transformation conceals most local, featural (e.g., eyes, nose, mouth) and relational information (e.g., reliable spacing between cardinal features; also termed “2^nd^-order relations”). Mooneyized pictures have been used to investigate (neuro-)cognitive processes [14], neurological diseases and developmental aspects of normal and psychopathological behaviour [15], and expertise-based face perception [16]. Doi et al. [12] found that 18 month-olds preferred upright Mooney faces to inverted ones, while 6 and 12 month-olds did not (yet) show any preference. In another study, Leo and Simion [17] reported that even newborns could detect Mooneyized faces as faces. Still, other studies show that further sophisticated processing of Mooney face, e.g. recognition, is quite unmatured in 6-8 year old children and progresses until 18-21 years of age [18]. In a study with pupils of the second to eighth grade (age about 7 to 13) Mooney [13] found a significant positive relationship between participants’ school grade and recognition performance for age categories and gender.

It has long been held that featural face processing develops in early infancy while configural and holistic face processing matures later until the age of about 10 years [19-22]. In a recent critical review of the literature, however, McKone et al. [9] found that even young children use all standard adult face recognition abilities. The authors concluded that “there is no qualitative chance in face perception beyond 4-5 years of age” (p. 474). This does not say that development of face detection and recognition is finished it only states that the structures and rules for most face processing stages already exist in early childhood. Only recently, it could be shown that under certain circumstances even the quantitative face recognition ability may be at adult level in early childhood [23]. Cortex specialization for face recognition emerges only gradually over the first decade of life, though, as fMRI studies have shown (for an overview see [24]). Of course, cortex development does not stop then, cortical networks keep changing all life [25]. Therefore, changes in face recognition capabilities can be expected to extend well into the adult age. In a very extensive internet-based study, Germine et al. [26] found that face learning abilities indeed peak not before the early thirties. This study, however, focused on specific face *recognition* issues tested by the “Cambridge Face Memory Test” (CFMT [27]). No comparable study exists for face detection and categorization.

**Figure 1:**
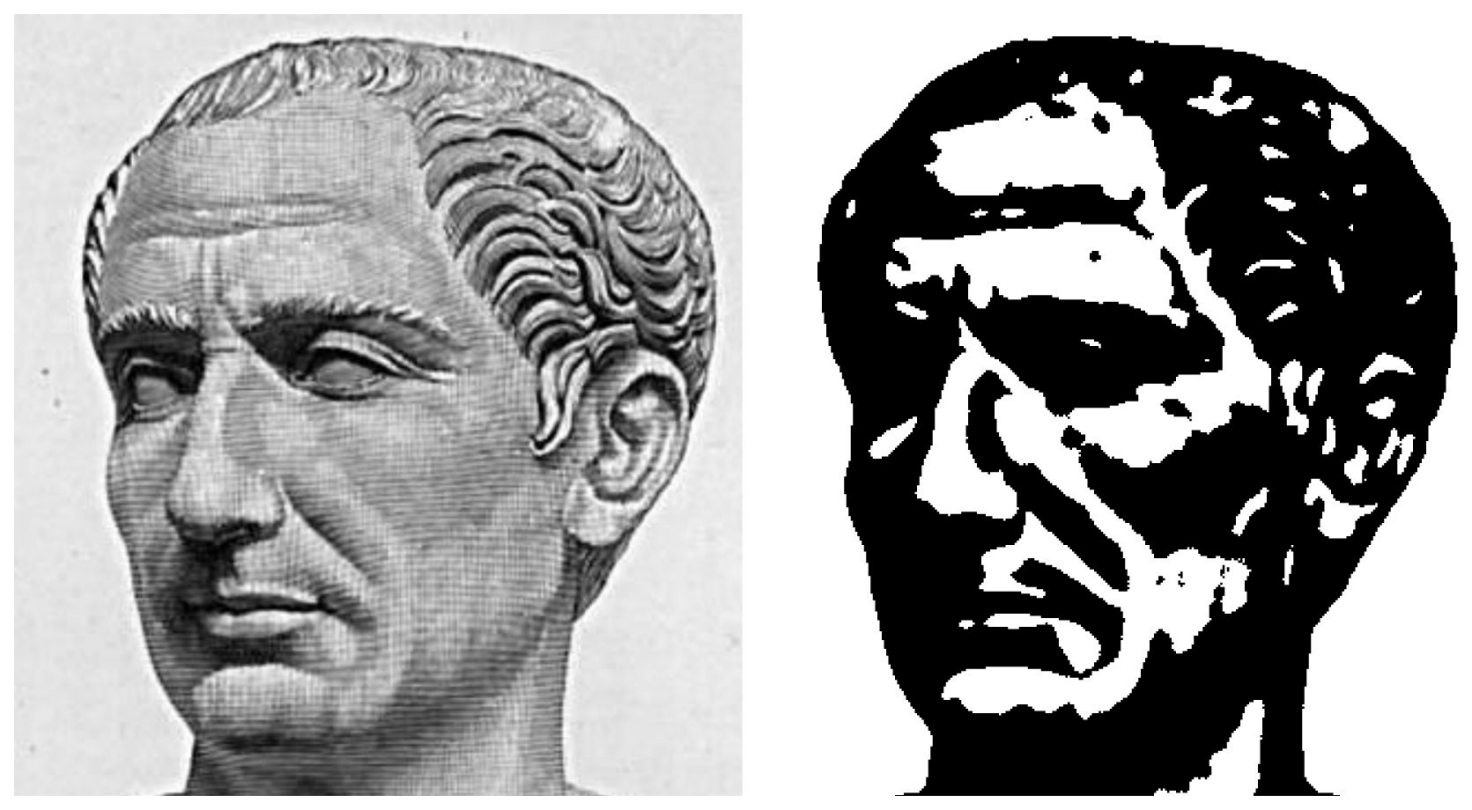
Illustration of a Mooney face based on the manipulation proposed by Craig M Mooney—note: this specific illustration is not part of the original set of Mooney faces and was not used in the present experiment. On the left (a), an original photograph of a bust of Gaius Julius Caesar (bust is situated in Naples National Archaeological Museum; the photo stems from the Wikimedia Commons), on the right (b), a two-tone (“Mooney”) image of it with high contrasts, which was previously smoothed by a Gaussian filter.

In order to shed some light on the development of face *detection* and face *categorization*, we employed high-contrast two-tone face images made of black and white providing only low residuary information and thus carrying face processing skills to the limits [13]. Despite this strong reduction of facial information, most people are still able to reconstruct from Figure 1b the image of a face, the angle of view, the gender, emotional expression and even identify it as a portrait of Julius Caesar—and such capabilities have been shown to be trainable [14]. If and only if such “Mooneyized” displays are recognized as faces, they activate the fusiform face area [2,28,29] which indicates face-related processing. Specifically, performance in any Mooney face test can be assumed to reflect *holistic* face detection skills. It does not correlate with performance in the Gollin Incomplete Figures Test for objects and the Poppelreuter overlapping figures test (see [30]). The fMRI results and Foreman’s study indicate that Mooney face detection and categorization does not primarily employ general closure abilities, but also rely on face-specific cognitive skills. A conceivable delay of several seconds is observed with Mooney face recognition. We may assume that in this time span the brain tries to match the sparse visual information with mental images of objects or faces. Given the neural network character of these stores, a sufficient similarity will generate a threshold potential. Due to the indeterminacy of Mooney pictures, several rivalling alternatives will we tested and discarded, before the target face is recognized. The missing information will be filled in from memory and the result will then be processed by the face processing network.

### The present study

If Mooney faces are processed by a face-related neural network, this network has to be extensively trained with faces at all angles and lighting conditions before it can interpret the relatively sparse data from a Mooney face. This would require a long training phase posing the question: at what age can we expect performance to peak? Intelligence research has hypothesized that some cognitive abilities peak early in adulthood (fluid intelligence) while others increase well into late adulthood because they rely on experience (crystallized intelligence) [31]—this dissociate concept of intelligence is even part of prominent intelligence scales (see [32]). Also, one may expect performance to be quite sensitive to any degradation of the neural network, for example resulting from old age. In the present study we used Mooney’s original material and oriented towards his original procedure to study holistic face processing in a large number of participants (*N*=312) aged between 2 and 88 years in order to provide an age-adjusted standard for further studies.

## Experiment

### Method

#### Participants

We recruited 312 participants (*M*_*age*_=37.9 yrs, range: 2-88 yrs; 184 female; see further details on the distribution of the ages in Table 1) who had no reported vision impairment or difficulties in recognizing faces.

**Table 1:** Correctness data (means plus *SD*s in parentheses) for the tasks on face detection and Mooney gender and age decision, split by the age of participants; the *N* of the given samples per age is displayed in extra columns.

Age	*N*	detection	gender task	age task
02-05	12	.825 (.380)	.506 (.221)	.485 (.198)
06-10	36	.956 (.205)	.629 (.129)	.566 (.103)
11-15	21	.988 (.109)	.846 (.076)	.781 (.096)
16-20	19	.976 (.152)	.832 (.083)	.750 (.117)
21-25	18	.979 (.143)	.850 (.045)	.785 (.060)
26-30	23	.979 (.142)	.874 (.068)	.778 (.073)
31-35	24	.985 (.120)	.874 (.053)	.782 (.065)
36-40	18	.972 (.164)	.865 (.074)	.801 (.058)
41-45	20	.946 (.226)	.829 (.108)	.742 (.089)
46-50	27	.973 (.162)	.824 (.057)	.766 (.062)
51-55	21	.955 (.208)	.814 (.092)	.754 (.070)
56-60	16	.956 (.205)	.817 (.089)	.769 (.071)
61-65	13	.956 (.206)	.848 (.098)	.765 (.094)
66-70	13	.952 (.215)	.756 (.139)	.675 (.112)
71-75	13	.985 (.123)	.794 (.093)	.740 (.102)
76-80	7	.914 (.280)	.700 (.221)	.661 (.211)
81-88	11	.830 (.376)	.605 (.196)	.570 (.203)

#### Stimuli

Stimuli consisted of the original set of 40 Mooney faces [13] which were presented on paper with a picture size of 4.7 × 3.1 cm (H × W). The whole set comprised 15 female faces, 25 male, 20 faces were “young” and 20 were “old”.

#### Procedure

Participants were asked to evaluate the face set in a pseudo-randomised sequence. First they decided whether or not the displayed stimulus was a face (“face detection task”: yes, no), then they evaluated gender (“gender decision task”: male, female) and age (“age decision task”: young, old). They were not allowed to revise their decision. In case they did not perceive any face-like object, we instructed them to omit the picture. If and only if they saw a face they were instructed to specify age (forced choice: young/old) and gender (forced choice: male/female). The procedure took about 10-15 minutes for each participant. Note: All children (approx. below 12 years of age) had an assistant, usually one of the parents, who supported the children, especially to explain the task verbally. For very young children, additional breaks were introduced as their attention was often diverted after a couple of trials. Nevertheless, even very young children were able to complete the task.

#### Ethical statement

After the experiment had ended participants were fully informed about the study and allowed to ask questions. Due to the simple and easy-to-conduct nature of the study, only verbal, unwritten consent was obtained from each participant prior to the experimental session; in the case of minors, caretakers gave their approval, again, by verbal consent. Persons who did not consent were not included in the study. For each participant our records indicate that verbal consent had been obtained. As all data was collected anonymously and no harming procedures were used, ethical approval (specifically for the present) was not sought for the execution of this specific study, but the experiment was part of a more extensive project of testing participants with facial stimuli for which the local ethics committee (“Ethikrat der Otto-Friedrich-Universität Bamberg”; dated 11 February 2011) gave an umbrella approval before any of the targeted studies were conducted.

## Results

**Figure 2:**
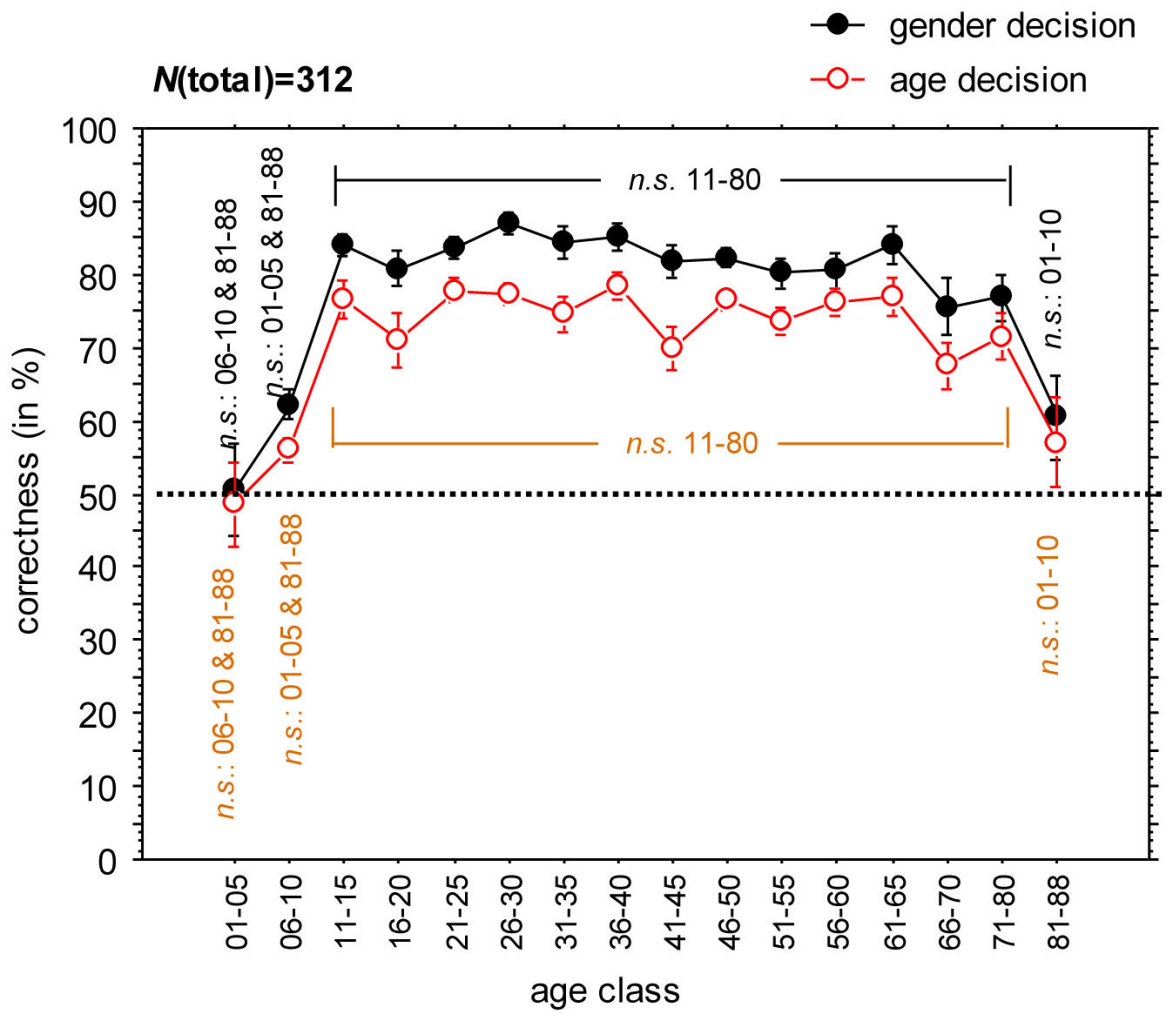
Correctness data split by age classes for the age (red, unfilled dots) and the gender (black, filled dots) decision task, respectively. Error cars indicate 95%-confidence intervals. Additional information given by “*n.s.*” show non-significant pairwise comparisons, e.g. the pairwise comparison between age group “01-05” was not significant when compared with “06-10” and “81-88”, meaning all *other* comparisons were significant at the Bonferroni adjusted level (see body text for more information on the alpha-error correction).

We found a high recognition rate for the face detection task (Table 1): Even in the youngest and oldest group we found rates higher than 82%. For the young up to middle-aged adults, the recognition rate exceeded 92% in all age groups. Gender and age assessment performance from Mooney faces increases up to about 15 years of age, and decreases from 70 years on (Figure 2 and Table 1). We determined the percentage of correct answers from the number of detected faces, not from the total number of Mooney pictures shown. Gender assessment from recognized faces was lower than expected. From the literature we know that based on original face photographs, gender recognition is nearly perfect in adults [33], and is about 80% correct in seven-year-old children [34]. With Mooney faces, the performance of the gender decision task was at chance level in the youngest group, and slightly above chance in the group from 6-10 years and in the oldest group. Even in the adult group, it did not exceed 85%.

Data analysis was based on three different variables: (a) rate of non-recognised faces in the face detection task, (b) correctness of age decision, and (c) correctness of gender decision. Data was assigned to age groups with a gradation of 5 years (e.g., 06-10, 11-15, etc.), with the exception of the lowest (02-05) and the highest age class (81-88).

### Mooney face processing from a developmental perspective

First, the data from the participants was analysed by 3 independent one-way factorial Analyses of Variance (ANOVA), with age of the participants as between-participants factor. Rate of non-recognised faces, correctness of age and gender decision were used as separate dependent variables. Concerning the rate of non-recognised faces (on average, only 5.7% of the stimuli were not detected as faces at all), there was a main effect of participant age, *F*(16, 295) = 3.22, *p* < .0001, η_p_^2^ = .149, with a strong decrease in the non-detection rate in the first 15 years of life, a plateau until about the age of 70 and a strong increase from about age 70 on. Similar, but even more pronounced effects were obtained for the age and gender decision task. We found a medium-to-large main effect of age of participants, *F*(16, 295) = 14.74, *p* < .0001, η_p_^2^ = .444, for the age decision task. As illustrated in Figure 2 (see also Table 1), there was a strong developmental component for the first 15 years with a massive increase in performance from approx. 50% to approx. 80%. The results for the gender decision task corresponded accordingly. The ANOVA revealed significant main effect of age of participants, *F*(16, 295) = 16.81, *p* < .0001, η_p_^2^ = .477. Again, there was a pronounced performance improvement within the first 15 years, then a plateau of performance up to 80 years, followed by a marked deterioration from 81 years on. Further pairwise comparisons of participant age categories were conducted for the age decision task and the gender decision task, respectively. Due to the number of possible comparisons for each age category, the α-level was Bonferroni-adjusted to .05/16 = .003125. For both the age decision and the gender decision task, we have calculated the significance levels for all pairwise comparisons documenting a fully consistent data pattern for both tasks, graphically shown in Figure 2. No significant effect was found until the age of 81 and above.

## General Discussion

In the present paper, we assessed face detection and categorization performance of 312 participants aged between 2 and 88 years. We asked the participants to decide whether they saw a face in a two-tone picture („Mooney face“) and if so, to identify gender and age of the faces. Face detection as well as gender and age categorization turned out to be age-dependent. For face detection, the results show a perfect detection of 95% beginning with the age group 6-10. For the face categorization tasks (gender as well as age), we revealed a steep performance increase up to about 15 years. There is a plateau until about 65 years with a steep and ongoing decline from 81 years on, though the ceiling effect in the detection rate may hide more subtle differences in this age range. For age and gender decisions, the pairwise comparison did not reveal significant performance changes between 11 and 80 years.

The large number of participants allowed us to set up an age-dependent performance chart that can be used as a reference table for age-related performance data for Mooney face tests.

From publications on gender recognition from facial photographs (e.g. [33]), we would have expected a better recognition rate for gender in all groups. There are several possible explanations for this phenomenon. Visual closure demands that a mental image of the face is formed and gender will be evaluated from this image. At an early age this image may be somewhat poorly defined and therefore the gender decision may be difficult. As not everyone has well-developed mental imagery [35], the precision of gender evaluation from mental imagery may be lower for all ages. And above all this, the participants may have expected to find a face in each Mooney picture they were shown. Given the inclination of the human visual processing system for *pareidolia* (seeing Gestalten in random or ambiguous stimuli, e.g., faces in clouds, the moon, or the bark of trees), the participants may have seen *some* face in the pictures, but not necessarily the one that was depicted. Because we asked the participants to only evaluate gender and age if they saw a face, erroneous face identification would result in a random gender and age assignment. This would, of course, bias the results towards the lower end. While the effect is certainly not negligible, the extent is difficult to assess. In a study with children and adults, Mooney [13] found that the participants tended to falsely recognize nonsensical black-and-white drawings as faces. Adults erroneously categorized 63% of nonsensical pictures as faces, while the percentage in children increased with their age from 54% at the second grade (about seven years) to 85% and more in the sixth to the eighth grade (about 11 to 14 years). Others, though, found much lower values for false positive identifications of nonsensical two-tone drawings. Uhlhaas et al. [18] reported less than 30% for all ages and Gruetzner et al. [36] merely 17.5% in adults. In our study, the excellent rate of correct gender categorization in the groups from 11 to 65 years (between 81.4% and 87.4%; see Table 1) does not leave much room for pareidolia. At earlier ages, the categorization results are not much better than chance. Even if there is some effect of pareidolia, the results are still surprisingly poor.

The basic face categorization mechanisms have been shown to be in place very early in life [9], but face processing under real world conditions is often based on very incomplete visual data. Faces must be detected and categorized under difficult lighting conditions and different viewing angles. These conditions conceal details, distort the real face, cover major parts of faces and strain the visual apparatus to its limits. This is not really reflected in typical face processing test that show perfectly lit frontal views of faces. The original Mooney faces, which we used, are also shown at different angles and require visual closure. Therefore, while all qualitative face processing mechanisms may be fully functional at 4 or 5 years, the detection and categorization of faces under real-life conditions involving visual closure matures somewhat later. Visual closure comprises more than just the completion of interrupted lines. A recent MEG study [37] suggested that when perceiving closure in Mooney faces, brain areas are activated that relate to the processing of three-dimensional structures from shading cues and associated with the activation of long-term memory templates. The neural networks involved can be expected to need long years of training to perfect their response. The fusiform gyrus only gets involved when the visual closure processing stage identifies the two-tone image as a face, indicating that mental images of faces are used to complete the missing parts. The perceived image of a face seems to be a combination of the picture processed from early visual fields and the mental image generated from memory.

The drop in holistic face performance at higher ages has been observed before [38,39] but cannot be fully explained by a general deterioration of cognitive and intellectual abilities in healthy people, which is known to proceed much slower [40,41]. An event-related potential study revealed that older people show delayed latencies for recognising faces, and even more important, that the distribution of respective potentials changes [42]. In line with this, a meta-analysis of functional neuroimaging experiments revealed a reduction of brain activity in memory-related brain areas accompanied by greater activity in the left prefrontal cortex during face recognition [43]. If we assume that the set of cognitive functions used for Mooney face processing shifts with age, a disproportionately strong loss of performance will be observed when the cognitive reserves are exhausted. In addition, eyesight tends to worsen at advanced age [44]. In the present study, participants confirmed that their eyesight was normal or corrected-to-normal, but in older people, deterioration may go unnoticed [45]. Therefore, the observed performance deterioration in the Mooney face test in older people may be caused by a combination of deteriorating eyesight and overall changes in cerebral function.

## Conclusions

We have shown that Mooney face recognition proficiency takes a long time to learn and decreases markedly from about the age of 81. While the basic processes for face processing under easy perceptual conditions are in place in early childhood, it takes about an additional decade of experience to perfect the abilities for face detection and categorization under difficult conditions. The long time span of peak performance may be upheld by shifting the cognitive functions employed for the task.
